# Estrogen Receptor Alpha Distribution and Expression in the Social Neural Network of Monogamous and Polygynous *Peromyscus*

**DOI:** 10.1371/journal.pone.0150373

**Published:** 2016-03-09

**Authors:** Bruce S. Cushing

**Affiliations:** Department of Zoology, University of Maryland, College Park, MD, United States of America; University of Missouri, UNITED STATES

## Abstract

In microtine and dwarf hamsters low levels of estrogen receptor alpha (ERα) in the bed nucleus of the stria terminalis (BST) and medial amygdala (MeA) play a critical role in the expression of social monogamy in males, which is characterized by high levels of affiliation and low levels of aggression. In contrast, monogamous *Peromyscus* males display high levels of aggression and affiliative behavior with high levels of testosterone and aromatase activity. Suggesting the hypothesis that in *Peromyscus* ERα expression will be positively correlated with high levels of male prosocial behavior and aggression. ERα expression was compared within the social neural network, including the posterior medial BST, MeA posterodorsal, medial preoptic area (MPOA), ventromedial hypothalamus (VMH), and arcuate nucleus in two monogamous species, *P*. *californicus* and *P*. *polionotus*, and two polygynous species, *P*. *leucopus* and *P*. *maniculatus*. The results supported the prediction, with male *P*. *polionotus* and *P*. *californicus* expressing higher levels of ERα in the BST than their polygynous counter parts, and ERα expression was sexually dimorphic in the polygynous species, with females expressing significantly more than males in the BST in both polygynous species and in the MeA in *P*. *leucopus*. *Peromyscus* ERα expression also differed from rats, mice and microtines as in neither the MPOA nor the VMH was ERα sexually dimorphic. The results supported the hypothesis that higher levels of ERα are associated with monogamy in *Peromyscus* and that differential expression of ERα occurs in the same regions of the brains regardless of whether high or low expression is associated with social monogamy. Also discussed are possible mechanisms regulating this differential relationship.

## Introduction

Estrogen receptor alpha (ERα) plays a critical role in the expression of male social behavior [1,2]. Comparative studies of closely related rodent species have shown that the distribution of ERα within the limbic system is associated with mating strategies and levels of male prosocial behavior. In microtines (Microtus), prairie (*Microtus ochrogaster*)[3], pine (*M*. *pinetorum*)[4], and Mandarin voles (*M*. *mandarinus*)[5], and dwarf hamsters (*Phopodus sp*) high levels of male prosocial (positive affiliative) behavior [3], which includes the formation of long-term pair bonds and parental care, is associated with low levels of ERα in the medial amygdala (MeA) and/or the bed nucleus of the stria terminalis (BST)[3–6]. This is in contrast to closely related polygynous species such as montane voles (*M*. *montanus*) [4] and unrelated species such as rats [7,8] and mice [9] where both males and females display high levels of ERα in the MeA and BST. Even within species there is a strong inverse correlation between male prosocial behavior and ERα. In prairie voles, the classification of social monogamy (see below) was based upon the behavior of prairie voles in Illinois [10,11]. In contrast males from Kansas display significantly lower levels of prosocial behavior and higher levels of ERα in the MeA and BST than males from Illinois [4]. Neonatal castration of male prairie voles disrupts subsequent expression of male prosocial behavior and the ability of vasopressin to stimulate pair bonds [12], and this is associated with over-expression of ERα in neonatally castrated adult males [13]. Finally, viral vector enhancement of ERα has provided direct evidence for the role of ERα in males, as increasing ERα expression in the MeA [14] and the BST [15] significantly reduced the expression of male prosocial behavior in prairie voles.

Social monogamy is classified as a suite of behaviors, not sexual exclusivity, that include pair bonding, biparental care and low levels of male aggression [11,16,17]. It has been hypothesized that the evolution of high levels of male prosocial behavior is associated with a decrease in male aggression, as high levels of male aggression would conflict with male parental care [18]. In voles male aggression is correlated with ERα expression, with male prairie voles displaying lower levels of aggression than montane [19] and meadow voles (*M*. *pennsylvanicus*) [20], and Illinois male prairie voles are less aggressive than Kansas males [21]. Male ERα knock-out mice display little or no aggression compared with wild-type males [1]. In dwarf hamsters male aggression is seasonal. During the breeding season or on long day cycles, when males are highly social, they display lower levels of aggression and lower levels of ERα, while males on a short day cycle are typically more aggressive and there is a significant increase in ERα in the MeA and BST [22].

Although the above studies and logic make a strong case for the hypothesis that estrogen decreases or inhibits male prosocial behavior the question remains if this is the “universal” pattern associated with social monogamy. This may be particularly relevant in *Peromyscus* where studies indicate that both testosterone and estrogen facilitate male prosocial behavior and different patterns of male aggression. In the socially monogamous California mouse (*P*. *californicus*) estrogen enhances male parental behavior [23] and the onset of male parental behavior is correlated with a significant increase in aromatase, the enzyme that converts testosterone to estradiol [24]. Additionally, increasing testosterone levels in response to social interactions facilitated the expression of parental behavior. Following courtship males with higher testosterone levels were more likely to approach pups [25]. The expression of male aggression also differs in *Peromyscus* with the polygynous white-footed mouse (*P*. *leucopus*) displaying lower levels of aggression than the monogamous California mouse [26]. In repeated aggression tests male California males display a winner effect, with males becoming increasingly dominant after winning an aggressive encounter while this does not occur in white-footed mice [26]. However treatment with testosterone stimulated a winner effect in white-footed males, suggesting they are both capable of responding to steroids and that higher steroid levels are associated with increased aggression [27]. *Peromyscus* (both white-footed and *P*. *polionotus*, the beach mouse) are similar to dwarf hamsters in that males display much higher levels of aggression during short days, which is associated with increased ERα expression [28]. While these studies support the hypothesis that ERα expression is critical in the regulation of male social behavior in *Peromyscus*, they suggest that the role of ERα in regulating male prosocial behavior may differ from dwarf hamsters and microtines. Therefore, the goal of this study was to determine the pattern of ERα expression in *Peromyscus* based upon reproductive strategy and if they are the same or differ from other socially monogamous species. This was done using immunocytochemistry to examine ERα expression in two polygynous species, the white-footed mouse and deer mouse (*P*. *maniculatus*), and two socially monogamous species the California mouse [29,30] and the beach mouse (*P*. *polionotus*)[31,32].

## Materials and Methods

### Animals

*Peromyscus* were purchased from the Peromyscus Genetic Stock Center (University of South Carolina, Columbia, SC) and shipped to the University of Maryland. All animals were individually housed, sexually naive adults. Animals were maintained on a 14:10 h light/dark cycle and provided food (Purina Rat Chow) and water *ad libitum*. Brains were collected from males and females of each species (*P*. *maniculatus*, BW stock, *n* = 7 males, 6 females; *P*. *leucopus*, LL stock, *n* = 5 males, 5 females; *P*. *californicus*, IS stock, *n* = 5 males, 4 females; *P*. *polionotus*, PO stock, *n* = 6 males, 6 females). To avoid complications associated with estrus, vaginal lavages were performed on females (for method details see [3]) to determine stage of estrus and tissue was collected only during diestrus. All procedures reported in this study were approved by the University of Maryland IACUC prior to any research being conducted and were within the guidelines established by the National Institutes of Health Guide for the Care and Use of Animals.

### Localization of ERα immunoreactivity

**Fig 1:**
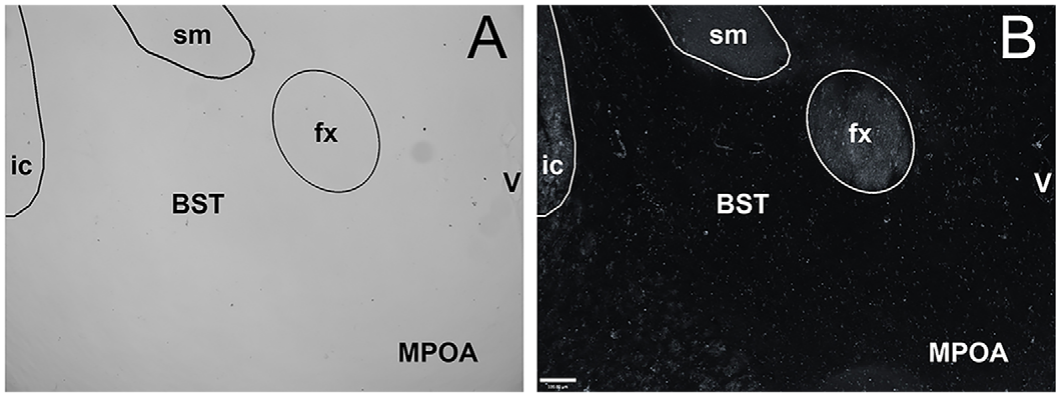
Bright field (A) and dark field (B) images of the same section taken at 10x showing the lack of specific staining in the bed nucleus of the stria terminalis (BST) and medial preoptic area (MPOA) following omission of the primary antibody. fx, fornix, sm, stria medullaris, ic, internal capsul, and V, third ventricle. Scale bar in B represents 100 microns.

Mice were deeply anesthetized with a mixture of Ketamine (67.7 mg/kg) and Xylazine (13.33 mg/kg) prior to transcardial perfusion with 4% paraformaldehyde and 2.5% acrolein in 0.1 M potassium phosphate buffered saline (KPBS; pH 7.6). Brains were removed and stored in 25% sucrose at 4°C until sectioned at 30 μm using a freezing sliding microtome. Sections were stored in cryoprotectant at −20°C until processed using standard Vector ABC immunocytochemistry (ICC) staining for ERα. Sections were rinsed in 0.05 M KPBS and then incubated at room temperature in 1% sodium borohydride for 20 min to neutralize acrolein and rinsed in KPBS, before being incubated in rabbit ERα polyclonal antibody (anti-ERα C1355, Millipore) at a concentration of 1:100,000 in 0.05 M KPBS +0.4% Triton X for 1 h at room temperature and then for 48 h at 4°C. This antibody binds to both free and bound receptors [33], reducing variation in staining due to potential differences in circulating hormone levels, and has been previously validated in *Peromyscus* [28,34]. Following incubation in the primary antibody sections were rinsed in KPBS and then incubated for 1 h at room temperature in biotinylated goat, anti-rabbit IgG (Vector Laboratories, Burlingame, CA) at 1:600 dilution in KPBS + 0.4% Triton X. Sections were then rinsed in KPBS and incubated for 1 h at room temperature in an avidin—biotin peroxidase complex (Vectastain ABC kit-elite pk-6100 standard, 4.5 μl A and 4.5 μl B per 1 ml solution, Vector Laboratories) in KPBS + 0.4% Triton X. Sections were rinsed in KPBS followed by rinses in 0.175 M sodium acetate. ERα was visualized by incubation in a nickel diaminobenzidine chromogen solution for 15 min. Lastly, sections were rinsed, dehydrated in alcohol, and mounted to slides using Histoclear and Histomount. To assure that staining was associated with the primary antibody the above protocol was followed absent the use of the primary antibody (Fig 1).

### Image analysis

Slides were coded and images captured on a PC and analyzed using IPLab (Scanalytics, Fairfax, VA) by an experimentally-blind scorer. IPLab generates a segment layer based upon intensity of the staining and a minimum contiguous pixel area representing a single “cell”. Only cell that were darker than background are segmented and regions larger than 10 pixels considered represented stained cells. Number of cells are counted by the program within the region of interest (ROI). Specific regions/nuclei were determined using established landmarks and the Paxinos and Watson rat atlas [35]. Image analysis was used to determine the number of cells expressing ERα-IR in the medial preoptic area (MPOA), BST posterior medial division, arcuate nucleus (ARC), ventromedial nucleus of the hypothalamus (VMH), and MeA posterodorsal. These areas were chosen because they are involved in a variety of aspects of reproduction and social behavior. One third of the total brain sections for each animal were stained. This was done to assure that matched regions were available from each individual [3,4,34]. The matched representative section containing the nucleus/area of interest was counted bilaterally and then cell counts for each hemisphere were averaged. It should be noted that volume of the region of interest was not measured, so that differences reported could be associate with relatively more cells in a region. These methods are published and have been previously established in assessing ERα immunoreactivity in *Peromyscus* [34] as well as *Microtus* [3] and dwarf hamsters [4].

### Statistics

Because ERα was predicted *a-priori* to be sexually dimorphic between species differences were analyzed separately for each sex by brain region using a one-way ANOVA [3–5,13]. If the ANOVA was significant, a Fisher’s PLSD was used to make post-hoc pair-wise comparisons. Within species between-sex differences were analyzed for each brain region using a *t*-test. Differences were considered significant if P < 0.05.

## Results

### Between Species

**Fig 2:**
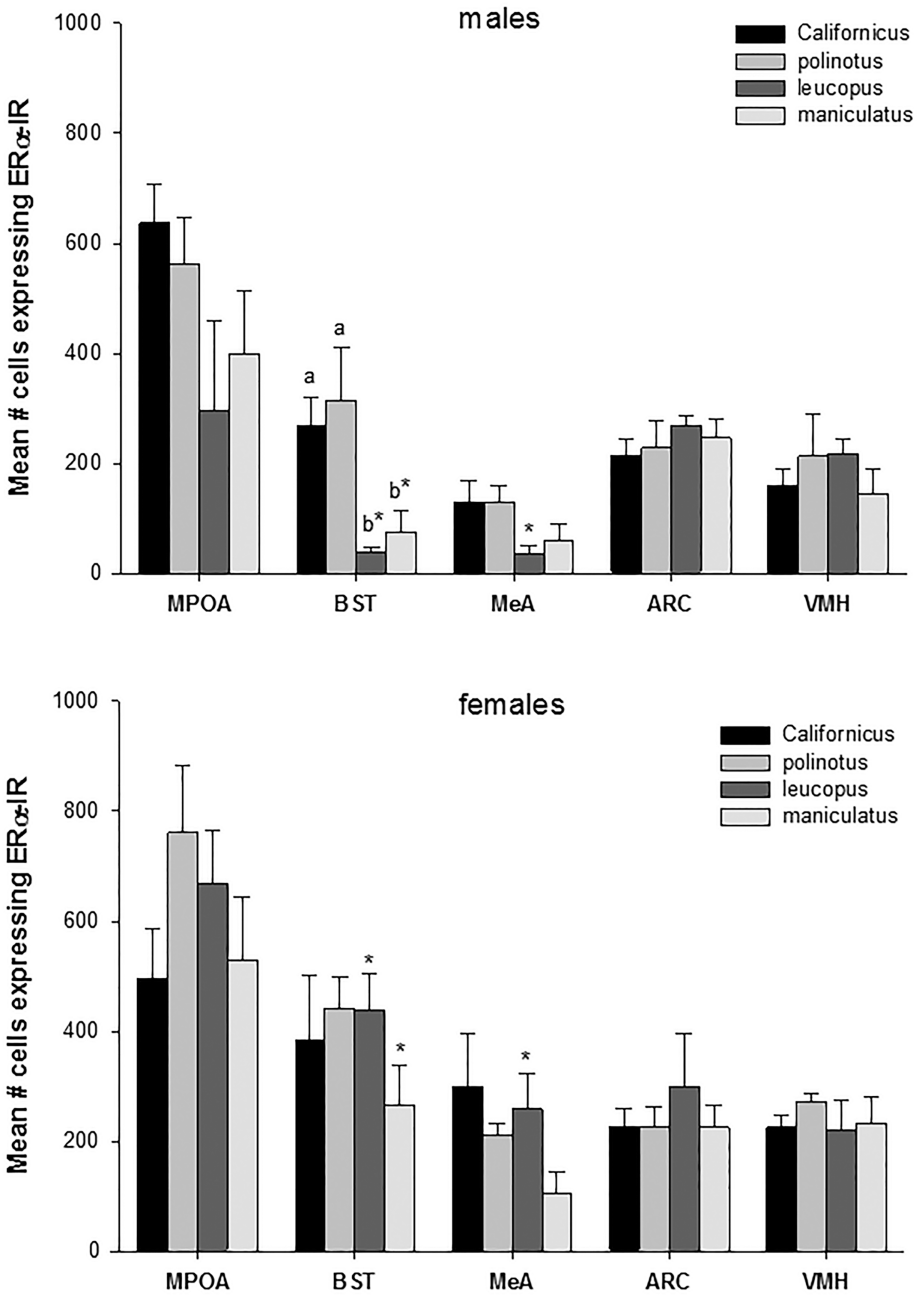
Shows the mean expression of ERα-IR by region in male and female *Peromyscus*. Polygynous males, *P*. *leucopus* and *P*. *maniculatus*, displayed significant higher levels of ERα-IR in the BST than socially monogamous males. Within species ERα-IR was sexually only in polygynous species with females displaying significantly higher levels in the BST than males in female *P*. *leucopus* more than *P*. *leucopus* males in the MeA. * = significant within species difference P < 0.05; different letters indicate significant between species within sex differences by region P < 0.05.

**Fig 3:**
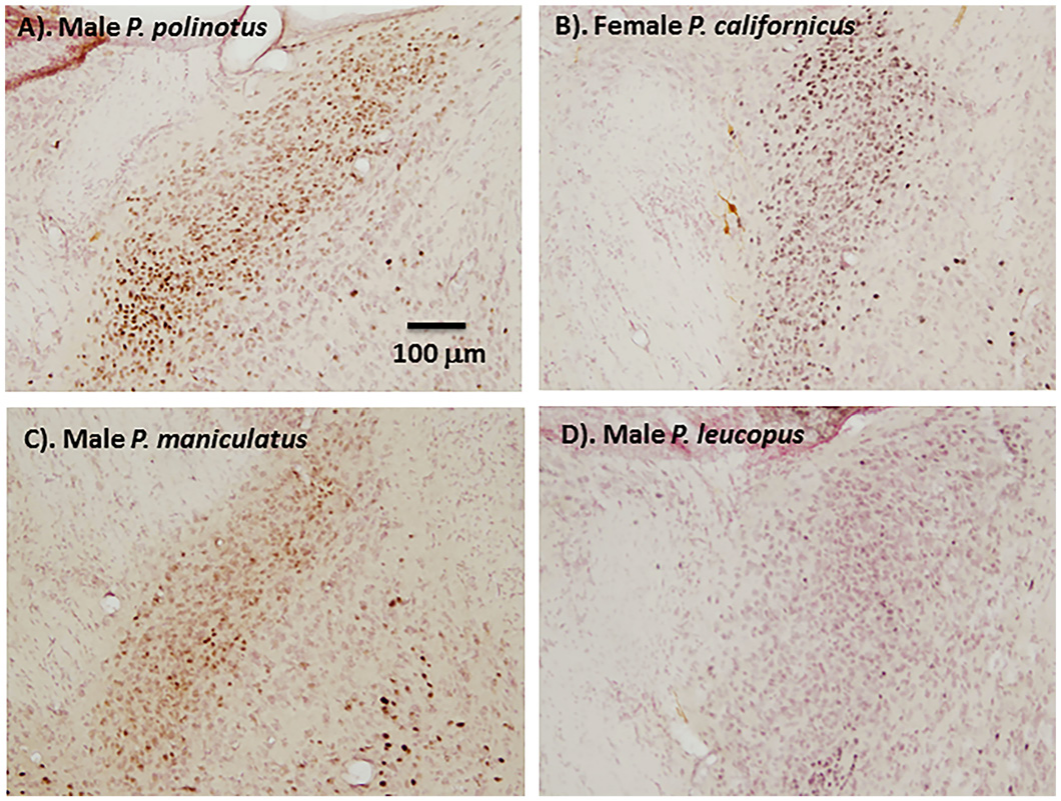
Representative photomicrographs (100x) of BST in male (A, C, D) and female (B) *Peromyscus*.— = 100μm.

There was a significant difference between males in the expression of ERα in the posterior medial division of the BST (F_3,17_ = 4.5, P < 0.05). The difference was due to significantly lower ERα-IR in white-footed mice and deer mice compared with both beach mice and California mice (P < 0.05 Figs 2 and 3). There was no significant difference in other regions or between females (Fig 2).

### Within Species—sexual dimorphism

ERα-IR was sexually dimorphic in the polygynous species. Male white-footed mice expressing significantly fewer ERα-IR cells than females in BST posterior medial and posterior MeA posterodorsal (*t*_*7*_ = 5.2, P < 0.01, *t*_*7*_ = 3.8, P < 0.01, respectively) and deer mice males significantly less in the BST (*t*_*9*_ = 2.4, P < 0.05).

## Discussion

As predicted, male ERα-IR expression was correlated with mating strategy and male social behavior. Socially-monogamous male California and beach mice displayed significantly higher levels of ERα-IR in the BST than their polygynous counter parts. ERα-IR was sexually dimorphic in the BST and/or MeA of the polygynous deer and white-footed mice, with females expressing significantly higher levels than males. Finally, ERα expression in the VMH and MPOA in *Peromyscus* differed from that reported in most other rodents. In rats, mice, voles, and dwarf hamsters ERα expression is sexually dimorphic with females expressing higher levels than males [4–9], while in all four *Peromyscus* ERα expression in the VMH and MPOA was not sexually dimorphic.

There are three potentially important points associated with these findings. First, the differential expression of ERα between polygynous and monogamous species supports the behavioral studies in *Peromyscus* indicating that greater sensitivity to estrogen, through greater ERα expression, is at least in part responsible for high levels of male prosocial behavior and aggression [23,28]. Second, that the brain regions in which ERα differs between males of closely related polygynous and monogamous species is consistent across rodent genera. Finally, and perhaps most informative, is that the results suggest that natural selection has acted differentially on the same underlying mechanism(s) to produce convergent behavioral evolution.

### Male Prosocial behavior

Although high levels of male prosocial/parental behavior have typically been inversely correlated with gonadal steroid levels in most rodent [36] and humans [37] empirical studies using *Peromyscus* suggest a positive correlation. In the socially monogamous California mouse high levels of prosocial behavior, especially parental behavior, are associated with high levels of estrogen/aromatase activity [23,24]. Seasonal changes in male *Peromyscus* behavior are correlated with changes in ERα expression [28], and estrogen and aromatase activity enhance male prosocial behavior [23,24]. A positive correlation between gonadal steroids and male prosocial behavior may not be limited to *Peromyscus*, as male Volcano mice (*Neotomodon alstoni*) displayed increase parental care when T levels were relatively high [38].

### Aggression

Although studies on the role of estrogen and ERα in male aggression have produced mixed results, when a direct relationship has been shown ERα is typically associated with increased aggression [1,39]. This includes socially monogamous microtines, dwarf hamsters [22], and *Peromyscus* [28]. “Seasonal” changes in ERα are associated with higher levels of aggression. In monogamous dwarf hamsters aggression occurs primarily during the non-breeding season, triggered by short day length and region-specific increases in ERα expression [22]. The polygynous white-footed mouse also displays increased aggression during short days [28]. In contrast male prairie voles only show high levels of aggression following the initial bond formation and mating with a female [17]. This very selective mate guarding may have evolved because following the initial mating, female prairie voles are sexually receptive and mate during delivery, “partum” receptivity, which occurs in the nest eliminating the need for mate “guarding” during subsequent receptive periods. If high levels of ERα expression are necessary for the expression of aggression then this may explain, at least in part, why selection has acted differentially in the California mouse. In contrast to prairie voles, female California mice undergo a more typical “post-partum” estrus that occurs 48 hr after birth and frequently seek extra-pair copulations [40]. In the field, female *Peromyscus* are more active and significantly more likely to be trapped when in estrus, while female voles are significantly less likely to be captured in estrus [41]. Therefore, male California mice may need to display high levels of male/male aggression to defend their territory and mate, while also displaying high levels of bi-parental care. This means that the selective forces acting on monogamous *Peromyscus* and voles are significantly different and means that California mice contradict the hypothesis that the evolution of social monogamy involves both an increase in male prosocial behavior and a decrease in male aggression [19].

### The role of the BST and MeA

Although prosocial behavior and aggression are regulated by complex neural circuits the differential expression of ERα associated with mating strategy appears to be limited to the BST and the MeA. The BST and MeA are derived from the same embryonic brain regions with the BST considered to be part of the extended amygdala and there are efferent and afferent neural connections between the two nuclei [42,43]. The BST and MeA are the initial regulators of many social interactions and are among the first regions to show neuronal activation during social contact, as they receive direct neural stimulation from both the olfactory and accessory olfactory bulbs [44–47]. They have been directly implicated in the regulation of a variety of social behaviors, including parental behavior [48,49], the formation of male-female pair bonds [14,50,51], and aggression [52,53]. In California mice, lesions of the amygdala disrupted parental behavior in males but not females [54]. In addition, these regions are sexually dimorphic in polygynous *Peromyscus* and monogamous microtines and dwarf hamster [3–6] suggesting that they are playing an essentially different role in males and females. Taken together the relationship of the MeA and the BST to critical sensory input, response to social cues, and connections with other critical regions of the brain means that differential expression of essential receptors, such as ERα, within either or both regions would have a major impact on the expression of social behavior [14,15].

### Mechanisms regulating Prosocial Behavior

The fact that both high and low levels of ERα are associated with high levels of male prosocial behavior raises the obvious questions of how and why differential expression produces convergent behaviors and mating strategies. This question and the possible answer represent the potential for significant advancement of our understanding of the regulation of male social behavior and should stimulate empirical studies. Many of the studies using voles (*Microtus sp*.) have focused on the role of vasopressin in males, V1a receptor expression, and/or variation in microsatellite length of the V1aR gene (for review see [55]). However this relationship does not exist in many other rodent taxa, and in fact it has been observed that outside the genus Microtus there is no correlation between microsatellite length and mating strategy [56]. In *Peromyscus*, the lack of a relationship between male social behavior and V1aR microsatellite length has led to the conclusion that additional mechanisms must be involved [57].

It has been hypothesized that the expression of social monogamy is, at the very least, an interaction between steroids and neuropeptides [13], as gonadal steroids play a major regulatory role in the effects of vasopressin and oxytocin. Social recognition, an essential aspect of long-term pair bond formation, has been hypothesized to be the product of an estrogen-dependent four-gene interaction, consisting of vasopressin, oxytocin and estrogen receptors [58]. In male mice it has recently been suggested that social disorders, such as autism and schizophrenia, which are characterized by an inability to bond and display affiliative behaviors, are also the product of an interaction between estrogen receptors and gene expression of vasopressin and oxytocin [59]. This may explain why, when studied independently, gonadal steroids often regulate many of the same behaviors as vasopressin and oxytocin (for review see [13,58,59]). Therefore, a logical extension of these two hypotheses is that social monogamy is also the result of the interplay of steroids and neuropeptides. If social monogamy is the result of a dynamic interaction of these factors then, while changing one may disrupt the expression of social behavior, changing more than one in a coordinated fashion could produce the same suite of behaviors.

This hypothesis is supported by studies in the socially monogamous prairie vole (*M*. *ochrogaster*) where neonatal castration eliminated the expression of adult male alloparental behavior [60], the formation of pair bonds [12] and disrupted the ability of centrally administered vasopressin to stimulate the formation of a partner preference [12]. Neonatal castration did not alter V1aR expression [12], but did produce a significant over-expression of ERα in the BST, MeA, MPOA, and VMH in adult males [61]. Direct manipulation of increasing ERα in male prairie voles inhibited prosocial behavior [14,15], suggesting the possibility that increasing ERα may have inhibited the response to vasopressin. However, if V1aR expression had also been manipulated then the effect could have been different. This concept is supported by the previous findings on the expression of ERα and AVP in the PVN of *Peromyscus* sp. In the species used in this study there was no correlation between ERα or AVP in PVN and mating strategy [34]. However there was an inverse relationship between number of cells in the PVN expressing ERα and AVP [34], suggesting that differential patterns of expression could be associated with the production of similar prosocial behavioral patterns.

In conclusion, ERα expression in *Peromyscus* varies based upon mating strategy, supporting the hypothesis that ERα plays a critical role in the expression of male mating strategies and prosocial behavior. Further, the results indicate that the BST and/or MeA are critical regions in the regulation of mating strategy and male prosocial behavior. However in *Peromyscus*, in contrast to previously studied rodents, it is the monogamous males that express higher levels of ERα while ERα is sexually dimorphic in the polygynous species. Finally, the results indicate that to fully understand the regulation of mating strategies it is necessary to study the mechanisms that regulate prosocial behavior and aggression in concert rather than independently.
